# Increasing urban health awareness in adolescents using an interactive approach: evidence from a school-based pre-post pilot study in Rome, Italy

**DOI:** 10.1186/s12889-023-15778-6

**Published:** 2023-05-11

**Authors:** Doris Zjalic, Alessio Perilli, Lorenza Nachira, Teresa Eleonora Lanza, Giuseppe Santoli, Andrea Paladini, Walter Ricciardi, Chiara Cadeddu

**Affiliations:** 1 Section of Hygiene, Department of Life Sciences and Public Health, Università Cattolica del Sacro Cuore, Rome, Italy; 2 Italian Institute for Planetary Health, Rome, Italy

**Keywords:** Interactive education, Climate change, Youth, Planetary health

## Abstract

**Background:**

Cities contribute to and are affected by the climate crisis, determining significant health issues in urban settings. Educational institutions have a privileged position to contribute to achieving the transformations needed for a healthier future, so Urban Health education is fundamental to empowering the health of the youth living in cities. This study aims to measure and raise the awareness of Urban Health among students attending a high school in Rome (Italy).

**Methods:**

An interactive educational intervention, consisting of four sessions, was conducted in a Roman high school during spring 2022. Overall, 319 students aged between 13 and 18 attended the sessions and were asked to complete a 11-items questionnaire before and another after the interventions. Data was gathered anonymously and analyzed using descriptive and inferential statistics.

**Results:**

Fifty-eight percent of respondents improved their post-intervention questionnaire score, while 15% did not improve and 27% got worse. The mean score significantly improved after the intervention (*p* < 0.001; Cohen’s d = 0.39).

**Conclusions:**

The results suggest that school-based interventions using interactive approaches on Urban Health could be effective in increasing students’ awareness and promoting health especially in urban settings.

**Supplementary Information:**

The online version contains supplementary material available at 10.1186/s12889-023-15778-6.

## Background

Urban areas are currently the center of economic and social activities, accommodating over 55% of the world population (corresponding to 4.2 billion people), a percentage that is expected to increase to 68% by 2050 [1]. They provide access to a range of services and promote communication and integration, but also pose significant health risks to their inhabitants. Exposure to physical and chemical agents, mental and social stressors, and the impacts of the climate crisis are among the primary concerns and have a synergic effect on the health of urban populations. Notably, urban settlements themselves contribute to the climate crisis, further exacerbating the health risk [2, 3].

Human health depends strongly on the environment in which a person lives [4]. While the natural environment provides the concrete basis for human survival and development, it undergoes adaptation and transformation by humans. As a result, a complex interplay exists between the natural environment and the social environment, with each exerting an influence on the other and collectively defining the context in which individuals live. The quality of urban environments and its interactions with social, economic, and cultural factors have a significant role in determining the quality of life and health status of urban residents [5].

From these assumptions, Urban Health seeks to integrate health protection and promotion actions into urban planning, fostering the interaction between different disciplines such as medicine, urban planning and architecture [6]. One of the most effective Urban Health interventions is the implementation and improvement of urban green spaces [7], which could offer many environmental, social, and economic advantages, resulting in the co-benefits of helping mitigate the climate crisis, improving resiliency and recovery from its impacts, and improving human health and wellness [8]. The enhancement of local air quality, enjoyment, jobs, and habitat maintenance, the reduction of the urban heat island effect, and the promotion of physical activity are among the main effects of urban green spaces. The importance of nature and natural environments, such as green open spaces, community gardens, and picturesque vistas of nature, in promoting physical activity has become a topic of increasing interest in recent years. Promoting natural environments' significance for health through land-use planning has been argued since they offer healthy opportunities for people, such as to participate in physical activity [9–11].

Urban Health is essential to allow transdisciplinarity among professionals and can be a good basis for the increase of citizens' awareness of climate crisis-related problems. The World Health Organization (WHO) has recognized the challenge of urbanization and developed the Urban Health Research Agenda [12, 13], a set of global research priorities for 2022–2032 that is anticipated to have a medium- to long-term impact at both the global and subnational levels and will provide evidence for the design and implementation of multisectoral interventions that promote Urban Health [14].

Education in Urban Health is an essential step to empower the well-being of the youth who live in urban settings, as stated in the “Urban Health Rome Declaration” [15]. Therefore, educational institutions—as indicated in the “Planetary Health education framework”—could provide a pivotal contribution to the transformations needed to achieve a healthier future [16]. For all these reasons, it is extremely important to carry out in-school educational interventions concerning health in the urban context [16] and assess their performance.

Not all teaching approaches have the same capacity to improve knowledge. There is extensive evidence that an interactive approach is preferable to a standard teaching approach because it achieves greater learning effectiveness and trains life skills [17].

Our study aims to measure knowledge and awareness about Urban Health through a pre-/post- intervention questionnaire and an interactive educational intervention in a student population of a high school in Rome, Italy.

## Methods

### Study design and setting

A pre-post pilot study was carried out by a team of Public Health residents of the Università Cattolica del Sacro Cuore at the high school Liceo Scientifico Statale “Nomentano” in Rome, Italy. “*Liceo scientifico*”, literally translating as “scientific high school”, is a kind of high school designed to give students the skills to progress to any university or higher educational institution and emphasises the link between the humanistic tradition and scientific culture [18]. The “Nomentano” high school was chosen due to a previous connection of the teaching staff of the school with the lead researcher.

**Fig. 1:**
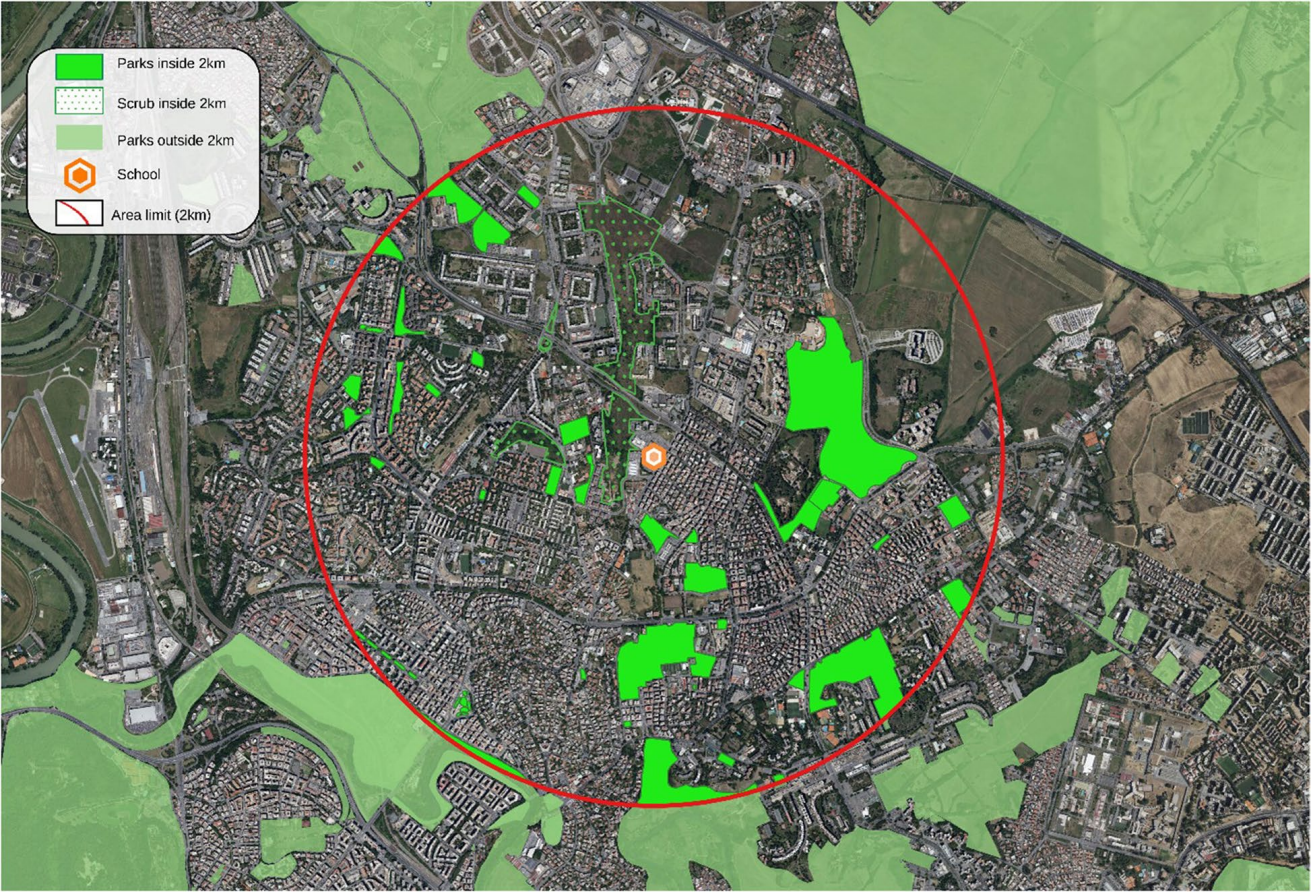
Map of parks and shrubland within a radius of 2 km from the school building

The school building is located outside the historic city center, in a neighborhood with a fair amount of public greenery, in particular 111 hectares of public parks (8,83% of total area) and 12 hectares of shrubland (0,95% of total area) within a radius of 2 km (1256,59 hectares) (Fig. 1). As can be seen from Fig. 1, outside the two-kilometers radius—but still in the vicinity -, there are several other green areas of considerable size [19].

The catchment area of the school is a large area, characterized by a multifaceted population with various socio-demographic backgrounds, mostly corresponding to two of the first-level administrative subdivisions of the Municipality of Rome, called *Municipi* (i.e. sub-municipalities): *Municipi* III and IV, where over 80% of students reside. The index developed by Lelo et al. 2021, based on urban quality, jobs density, education, cultural services, social relations, reveals a notable variability across the above *Municipi* [20]. In terms of economic conditions, the 2019 average income per person in *Municipi* III and IV amounts to 25.920,75 and 21.815,24 euros, respectively, slightly lower than the city’s average, which stands at 26.082,96 euros. Notably, each of central *Municipi* I and II features an average income that nearly doubles that of *Municipio* IV [21]. The 2011 social and material vulnerability index inside *Municipi* III and IV varies among their second-level administrative subdivisions, called urban zones (*zone urbanistiche*), ranging from 95,5 in the Sacco Pastore urban zone to 112 in the Tor Cervara urban zone. The index combines multiple vulnerability dimensions, i.e. education level, family structures, housing conditions, labor market participation and economic conditions and its average value in Italy is 100 [22].

### Intervention and study sample

The intervention consisted of four interactive sessions related to Urban Health, held weekly between March and April 2022, and its measure of effectiveness was estimated through the administration of a questionnaire before and after the educational sessions. All the school classes were invited to participate in the project: twelve of them joined, including 1st through 4th grade students; senior students, i.e., attending the 5th grade, were not included for overload study issues. Given the descriptive nature of the study, sample size was not determined in advance.

The educational sessions were structured based on the Planetary Health Educational Framework to allow interaction between the residents and the students and were conducted with the support of original PowerPoint presentations prepared after extensive literature searches, integrated and validated by the whole team and supervised by a senior researcher. According to the number of students per class, one or two residents in each class held the sessions made by small lessons, educational videos, role-playing games and time for collective discussion. Each session lasted approximately one hour.

The first session introduced the definition of climate change and the Anthropocene scenario. The second and third interventions focused on teaching the importance of Planetary Health and in particular Urban Health, the pivotal role of green spaces in the context of cities and their impact on human health. The fourth and last consisted of various teamwork games aimed at recalling what was learnt in the previous sessions. All the questionnaire topics were covered during the lessons.

### Survey design and Delphi validation

An email Delphi survey was conducted, inviting 10 experts in Urban Health. The Delphi method was used, which involves a systematic and interactive approach where a panel of experts answers questionnaires in multiple rounds [23]. Participants were asked to rate each question based on general relevance and coherence to the objective of the study through 5-point Likert items. The Content Validity Index (CVI) is calculated by dividing the number of experts whose score for each item was equal to or higher than 3 (3, 4 or 5) by the total number of experts involved. A CVI higher than or equal to 80% is considered indicative of the item inclusion in the questionnaire; a value between 70 and 79% is indicative of the need to review the item; a value lower than 70% is suggestive of the item removal.

In the first round, participants were asked to indicate their opinions on important topics to be included in the questionnaire, in light of the intended target population of the final survey. In the second round, 13 questions were developed by the researcher based on the domains that received a CVI higher than 80%. Additional domains were only included if they were listed by ≥ 15% of respondents. Comments from the first round were incorporated into the second-round questionnaires.

The response rate in the Delphi rounds was 100% in the first and 90% in the second. In the first round, 6 domains achieved a CVI of 100%. The researchers created a set of 13 questions related to the 6 topics agreed upon in the first round, i.e. the 11th Sustainability Development Goal, environmental and health benefits of green areas, air pollution, urban heat island, inequities and global urbanization. Each question had either five or four options, with one correct answer. Following the completion of round-two surveys, questions on inequalities and global urbanization were removed from the questionnaire due to difficulties in phrasing and negative feedback from respondents (VCI < 70%). Additionally, some questions were modified to incorporate feedback from respondents, which included suggestions to simplify language and shorten question length. The final questionnaire, resulting from the Delphi survey, consists of 11 multiple-choice questions: 2 with one correct answer out of 5 options and 9 with one correct answer out of 4 options.

### Data collection

Students were asked to answer an anonymous questionnaire before the beginning of the first lesson and after the end of the last one. The questionnaire resulting from the Delphi survey was administered as a web form on SurveyMonkey® and linked to a QR code that was shown to the students, who could scan it to complete the questionnaire. One point was assigned to each correct answer; zero points were assigned to any incorrect answer. Pre and post data were paired by assigning each student a unique identification code, which was unknown to the residents.

### Data analysis

The internal consistency of the questionnaire was evaluated by Cronbach’s alpha [24] and McDonald’s Omega, both in pre and post scores [25].

Descriptive statistics, including means, standard deviations (SD), medians and interquartile ranges (IQR) were calculated. Q-Q plots and histograms were employed to assess the variable distribution normality. In case of normality, Paired Student's T test was implemented to assess the significance of the difference between the scores that the students achieved before and after the interaction with the residents. Wilcoxon signed rank test was used when T test assumptions were violated. Effect size was provided as Cohen’s d and Cliff’s Delta, respectively. McNemar’s test was used to compare the proportions of right answers to individual questions. Cohen’s g was used as an estimation of effect size. Odds ratios for paired data with a 95% Confidence Interval (95% CI) were also calculated.

Unpaired Student's was used to assess the significance of the difference in scoring between male and female individuals of the population.

One-way ANOVA was employed to estimate the difference in scoring amongst each school year, the assumptions of normality were verified through QQ plots, while homoscedasticity of the data through Bartlett’s K-squared test.

Statistical significance was set at *p* < 0.05. All statistical analyses were carried out in R software, version 4.2.0 (CRAN®, R Core 2022) within the RStudio platform, version 2022.02.3 + 492 (© 2009–2022 RStudio, PBC).

### Ethical statement

This study was approved by the Ethics Committee of Fondazione Policlinico Universitario A. Gemelli—IRCCS (ID 4600).

The study was conducted in accordance with Good Clinical Practice, the Declaration of Helsinki, and EU Regulation 2016/679 (GDPR). Informed consent to participate in the study was acquired online prior to the administration of the questionnaire. Consent for data handling was not collected as the questionnaire only deals with anonymous data.

## Results

A total of 192 out of 319 students (60% of all participants) correctly completed the questionnaire both in March and in April. The reasons for exclusion of the 40% of participants were not having completed one of the two questionnaires, the non-response to all the questions, the non-correspondence of personal data by unique identification code and the presence of errors, such as age not corresponding to that expected. In relation to the gender of the sample, 35.4% of the sample students identified as “female”, 62.5% as “male” and 2.1% as “other”. At the time of the administration of the questionnaire, 47.9% of the students were attending the first year, 26.0% the second year, 10.9% the third year and 15.1% the fourth year. 52.6% of the students did not reside in the school neighborhood.

Fifty-eight percent of all main sample students obtained a higher score in April (after the interaction with the researchers), if compared to the score obtained in March. Fifteen percent of the main sample students totaled the same score both in March and in April and 27% had worse scores in April if compared to those of March. Cronbach’s alpha was 0.33 (95% CI: 0.18–0.46) in the March questionnaire and 0.48 (95% CI: 0.36–0.58) in the April one, whereas McDonald’s Omega was 0.57 and 0.71, respectively.

**Fig. 2:**
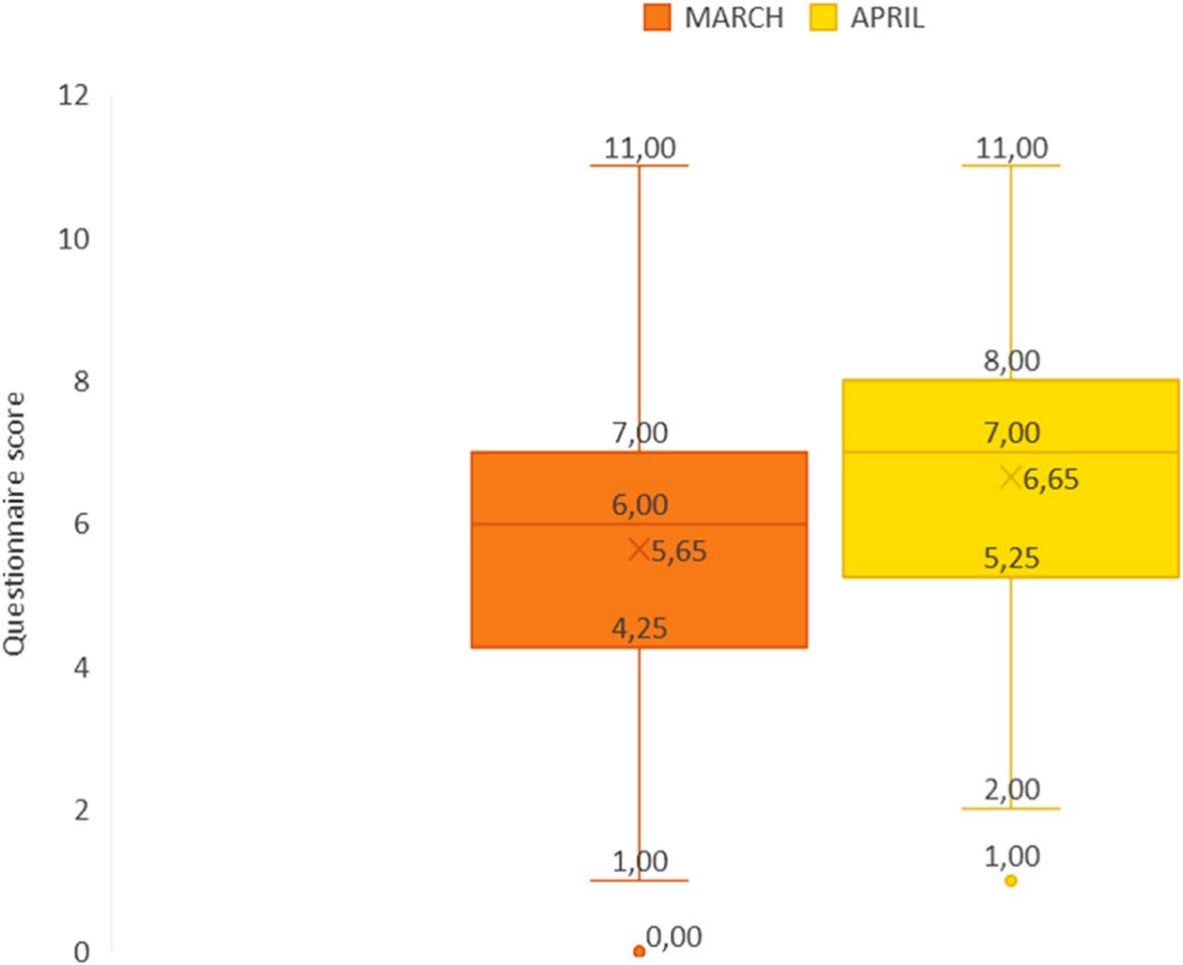
Scores obtained by the sample students in March and April

A statistically significant difference between scores attained in March and those in April was detected (*p* < 0.001), with a small effect size (Cohen’s d = 0.39; 95% CI: 0.24—0.54). The average improvement of the scores in the sample was 1.005 points (95% CI: 0.64—1.37) (Fig. 2). Stratification by year of study and gender shows similar results (Table 1). No significant association between the declared gender and the improvement after the interaction with the researchers was identified (*p* = 0.9).

**Table 1:** Comparison between the scores attained in March and April, overall, by school grade and gender

	**N**	**Mean (SD) pre**	**Mean (SD) post**	**Median (IQR) pre**	**Median (IQR) post**	***p*-value**	**Effect size (95% CI)**	**Effect size magnitude**
All	192	5.65 (1.8)	6.65 (1.97)	6 (2.25)	7 (2.25)	** < 0.001**	0.39 (0.24 – 0.54)	Small
**School Grade**								
1	92	5.37 (1.61)	6.41 (1.97)	5 (3)	7 (3)	** < 0.001**	0.38 (0.17—0.59)	Small
2	50	5.86 (2.15)	6.66 (2.17)	6 (2.75)	7 (2.75)	**0.03**	0.31 (0.03—0.6)	Small
3	21	5.86 (1.49)	6.9 (1.55)	6 (2)	7 (1)	**0.01**	0.42 (0.07—0.68)	Small
4	29	6 (1.89)	7.21 (1.84)	6 (2)	7 (3)	**0.02**	0.46 (0.07—0.85)	Small
**Gender**								
Male	120	5.48 (1.92)	6.5 (2.2)	6 (3)	7 (3)	** < 0.001**	0.37 (0.18—0.55)	Small
Female	68	5.93 (1.58)	6.9 (1.49)	6 (2)	7 (2)	** < 0.001**	0.43 (0.19—0.69)	Small
Other	4	5.75 (1.5)	7 (1.83)	6 (2.25)	7 (2.5)	0.31	0.61 (-0.59—1.91)	Medium

Significant values are highlighted in bold

A comparison between pre- and post-intervention results, with respect to individual questions, is reported in Table 2. A statistically significant difference was detected for items 2, 4, 5, 6 and 7 (*p* = 0.002; *p* = 0.009; *p* = 0.001; *p* = 0.04; *p* < 0.001, respectively).

**Table 2:** Comparison between the proportion of right answers to individual items before and after the intervention

Item	Topic	*p*-value	OR (95%CI)	Effect size (95% CI)	Effect size magnitude
2	11th SDG—general	**0.002**	1.88 (1.24 – 3.14)	0.15 (0.06 – 0.25)	Medium
3	11th SDG—specific	0.49	1.17 (0.77 – 1.85)	0.04 (-0.08—0.15)	Very small
4	Environmental benefits of green areas	**0.009**	2.12 (1.2 – 4.2)	0.18 (0.05—0.29)	Medium
5	Health benefits of green areas	**0.001**	2.07 (1.32 – 3.4)	0.17 (0.07 – 0.26)	Medium
6	Air pollutants	**0.04**	1.56 (1.02 – 2.46)	0.11 (0.01 – 0.21)	Small
7	Health benefits of green areas	** < 0.001**	3.42 (2.17 – 5.92)	0.27 (0.19 – 0.37)	Large
8	Urban Heat Island	0.09	1.57 (0.94 – 2.62)	0.11 (-0.01 – 0.23)	Small
9	Urban Heat Island and green areas	0.07	1.61 (0.97 – 2.82)	0.12 (-0.01 – 0.24)	Small
10	Biodiversity	0.85	1.07 (0.5 – 2.37)	0.02 (-0.18 – 0.19)	Very small
11	Green areas effects	0.16	1.38 (0.86 – 2.22)	0.08 (-0.03 – 0.19)	Small
12	Green areas soil benefits	0.82	1.05 (0.67 – 1.66)	0.01 (-0.1 – 0.12)	Very small

Significant values are highlighted in bold

**Fig. 3:**
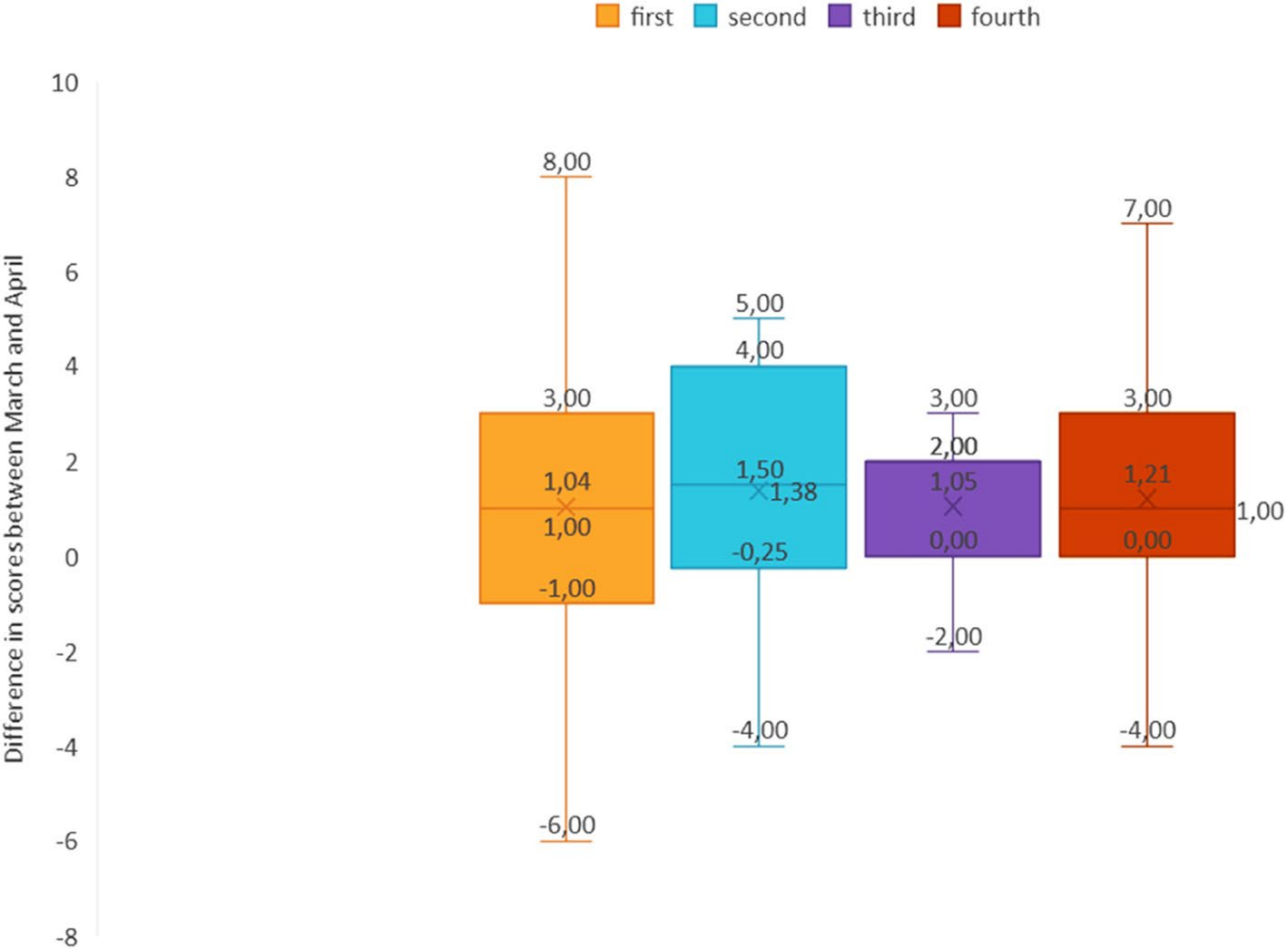
Difference in scores between March and April based on the school grade

The school grade attended by each student did not significantly affect the scores obtained, as shown through One-way ANOVA (*p* = 0.915) (Fig. 3).

## Discussion

The results of the present study suggest that school-based interventions using interactive approaches on climate crisis and Urban Health are effective in increasing the knowledge level on these topics and can be a useful way to promote Planetary Health in an urban setting, as 58% of students demonstrate an increase in score between March and April. In particular, a significant increase in final score was detected for those questions that included topics that were repeated several times, like the United Nations (UN) Sustainable Development Goals or the role of green areas in human health. This underlines the importance to structure interventions by frequently repeating the key messages of the argument.

The topics covered acquire increasing relevance in light of the current planetary issues [16, 26], as well as for the UN Sustainable Development Goals [27]. Indeed, rapid and dramatic changes in the climate scenario that affected the whole world last summer call on governments and the scientific community to further deal with the need to work not only for the mitigation of these changes, but also for adaptation [28]. There are many different approaches to increase adaptation and resilience of cities [29]. Some of these have immediate results, for example improving greenness to diminish the impact of heat islands. Education, instead, requires more time, but one that includes awareness, knowledge, skills improvement, values, and opportunities for participation does bring about in-depth learning and behavior change that can be essential for long-lasting adaptation of future citizens to climate change and to adopt pro-environmental behaviors that can contribute to mitigation [16, 30]. Knowledge is not the only skill needed to improve climate change awareness, but it is an essential component to allow future generations to make planet-oriented decisions, to live a healthier urban life and to ask politicians for healthier cities [31].

School-based health education has long been recognized as an effective prevention instrument, having been applied to a wide array of health issues and settings [32–35]. Italy has recognized how schools that promote health improve the knowledge of their students and have an impact on the entire community, actively acting to strengthen social capital and health literacy. For this reason, a document aimed at embedding "Health promotion" in the educational curriculum of schools of all levels, with a continuous and integrated approach, was approved in 2019 as a support to 2014–2018 National Prevention Plan (NPP) [36], and was referenced in the 2020–2025 NPP as the means for effectively integrate the school education and health systems [37].

The need to include Urban Health education in school curricula and to encourage the development of new programs to ensure the promotion of healthy lifestyles in cities is also advocated by the “Urban Health Rome Declaration” [15]. Our intervention accepts this invitation. In particular, it fits within the teaching already planned in Italian school curricula: “*Educazione civica*” (translating civics). With it, all students are guaranteed, each year, at least 33 h of activities aimed at promoting meaningful experiences that enable them to learn how to take care of themselves, others, and the environment, while fostering forms of cooperation and solidarity [38].

Interestingly, health interventions targeted to school students taking place in dedicated prevention centers were also reported in the literature, with positive accomplishments [39]. Interactive methods proved to be highly appreciated by young learners [40].

Previous research, with a similar approach to ours, investigated adolescents’ knowledge concerning One Health [41], which should likewise be regarded as a high-priority theme in educational interventions [42]. Nonetheless, to the best of our knowledge, this research is the first study worldwide that investigates the effectiveness of a school-based intervention on Urban Health knowledge in a student population, and it can be considered a pilot study for future research. The format of intervention is reproducible in different settings, and it can be easily adapted for different ages. Also, the intervention was onsite, allowing direct contact with students and their greater involvement. Nevertheless, the findings of this study must be seen in light of some limitations. The research was conducted on a small sample of students of one school only, thus affecting the generalizability of the results, and there is no control group, because of organizational issues related to identifying schools willing to participate in the study within the established timeframe. Another limitation of the study is that lessons were conducted by different residents in different classes and that may have caused bias in results due to different teaching styles. In addition, to assess internal consistency reliability, Cronbach’s alpha was used, which, despite being widely used in education science, still suffers from various intrinsic limits. Finally, it should be noticed that Cronbach’s alpha value in our study was not in line with the most adopted threshold for acceptability, although this should not be interpreted as indicative of an unsatisfactory tool [43]. To compensate for these limits, McDonald’s Omega was estimated, being regarded as a preferable alternative, considering its more feasible assumptions [44]. Furthermore, its value did reach acceptability.

Information provided by this study might benefit future interventions in properly spreading knowledge in this sphere, experimenting with innovative educational approaches and contributing to affecting behaviors and promoting much-needed cultural changes [45]. Further studies, with a robust, ideally experimental design and a larger sample, are strongly recommended.

## Conclusions

This study shows that interactive educational interventions may enhance Urban Health knowledge in a high-school student population. These findings may spur interest in the innovative approach put forward and eventually lead to similar interventions being carried out elsewhere, reaching a wider population, achieving widespread awareness and further accumulating evidence in this realm. The process may result in Urban Health education being embedded in school curricula, empowering students to promote their health and that of their families [46], with concurrent environmental benefits.

## Supplementary Information

**Additional file 1.**

## Data Availability

The datasets used and analyzed during the current study are available from the corresponding author on reasonable request.

## Abbreviations

WHO: World Health Organization
CVI: Content Validity Index
SD: Standard deviation
IQR: Interquartile range
95% CI: 95% Confidence Interval
GDPR: General data protection regulation
UN: United Nations
