# Supplementary material for: Increasing urban health awareness in adolescents using an interactive approach: evidence from a school-based pre-post pilot study in Rome, Italy
Source: BMC Public Health. 2023 May 11;23:855. doi: 10.1186/s12889-023-15778-6 (PMC10210429; doi:10.1186/s12889-023-15778-6)
Supplement: Supplementary file 1 — Additional file 1. [file 12889_2023_15778_MOESM1_ESM.docx]

URBAN HEALTH QUESTIONNAIRE

# Section A (general characteristics of students)

**Gender:**

M □ F □ I prefer not to answer □

**Age:** ____

**Nationality:** Italian □ Other □

**Name of your school:** ______________

**Educational course of study:__**____

**Course year:**  ________

**Postcode of residence:** __________

**Do you live in the same neighborhood as your school?**

Yes □ No □

# Section B (knowledge)

**The term 'urban green' is associated with different definitions, especially depending on the context in which it is treated. According to Istat sources, urban green means 'the heritage of green areas that insists on the territory of the municipalities managed, directly or indirectly, by public institutions such as municipalities, provinces, regions, the State. This includes different types of green areas: equipped green areas, urban parks, historic greenery, urban furniture areas and special areas, which include school gardens, botanical gardens, nurseries, zoos and other residual categories' (ISTAT, 2001).*

1. **Have you ever heard of Urban Health?**
   - Yes
   - No
2. **Goal 11 of the 2030 Agenda for Sustainable Development concerns:**
   - Clean water and sanitation
   - Good health and well-being
   - Sustainable cities and communities
   - Reduced inequalities
   - Decent work and economic growth
3. **Goal 11 of the 2030 Agenda for Sustainable Development envisages, by 2030, to:**
   - Provide access to safe, inclusive and accessible public green spaces, in particular for people in vulnerable situations
   - Reduce the negative environmental impact per capita of cities, in particular with regard to air quality and waste management
   - Provide safe, sustainable, and affordable transport systems for all, expand public transport, especially for women, children, people with disabilities and the elderly
   - All the above
4. **Green areas:**
   - Guarantee a good local microclimate
   - Attenuate the risk of floods
   - Determine the absorption of air pollutants (filtering action) and the reduction of noise levels
   - May have disadvantages such as reduced direct natural lighting of confined spaces
   - All the above
5. **The advantages of the presence of urban green areas for the health and well-being of residents consist in:**
   - Increased social relations
   - Greater contact with nature
   - Increased sense of general well-being of the individual
   - All the above
6. **Pollutants considered harmful to human health include:**
   - Particulate matter and tropospheric ozone.
   - Particulate matter, ground-level ozone, nitrogen dioxide and sulfur dioxide.
   - Particulate matter and tropospheric oxygen.
   - None of the above
7. **The frequent use of urban green areas has health benefits, such as:**
   - Reduction of chronic stress
   - Improvement in cognitive and social development
   - Reduction of certain health risks, such as obesity, cardiovascular and metabolic diseases
   - All the above
8. **The heat island:**
   - It is a thermal phenomenon characterized by important temperature differences between urban areas and peripheral/rural areas
   - It is a chemical phenomenon due to the presence of a watercourse that crosses the urban center
   - It is a phenomenon due to the greater presence of vegetation and bodies of water in an urban context
   - None of the above
9. **In contrast to the heat island phenomenon, green areas determine:**
   - An increase in temperature
   - An increase in greenhouse gas emissions
   - A cooling effect and microclimate regulation
   - An increased need for air conditioning
10. **What is biodiversity?**
    - The totality of all forms of life, the environments in which they live, as well as genetic diversity within species
    - The study of differences between animals
    - The totality of plants that live in an environment protected by Italian laws
    - None of the above
11. **The presence of urban greenery in the winter months:**
    - Mitigates the cold
    - Increases heat loss
    - Increases the thermal inversion effect
    - All the above
12. **The presence of plants:**
    - Prevents the absorption of rain in the soil
    - Reduces surface water erosion and hydrogeological risk
    - Increases the risk of flooding
    - None of the above
